# Extending the dynamic temperature range of Boltzmann thermometers

**DOI:** 10.1038/s41377-022-01028-8

**Published:** 2022-12-08

**Authors:** Thomas Pieter van Swieten, Jesse Merlijn Steenhoff, Auke Vlasblom, Ravi de Berg, Sam Pieter Mattern, Freddy Teunis Rabouw, Markus Suta, Andries Meijerink

**Affiliations:** 1 Debye Institute for Nanomaterials Science, Utrecht University, Princetonplein 1, 3584 CC Utrecht, The Netherlands; 2 Inorganic Photoactive Materials, Heinrich Heine University Düsseldorf, Universitätsstraße 1, 40225 Düsseldorf, Germany

**Keywords:** Optics and photonics, Optical materials and structures

## Abstract

Lanthanide-doped (nano)crystals are an important class of materials in luminescence thermometry. The working mechanism of these thermometers is diverse but most often relies on variation of the ratio of emission intensities from two thermally coupled excited states with temperature. At low temperatures, nonradiative coupling between the states can be slow compared to radiative decay, but, at higher temperatures, the two states reach thermal equilibrium due to faster nonradiative coupling. In thermal equilibrium, the intensity ratio follows Boltzmann statistics, which gives a convenient model to calibrate the thermometer. Here, we investigate multiple strategies to shift the onset of thermal equilibrium to lower temperatures, which enables Boltzmann thermometry in a wider dynamic range. We use Eu^3+^-doped microcrystals as a model system and find that the nonradiative coupling rates increase for host lattices with higher vibrational energies and shorter lanthanide–ligand distances, which reduces the onset temperature of thermal equilibrium by more than 400 K. We additionally reveal that thermometers with excited states coupled by electric-dipole transitions have lower onset temperatures than those with magnetic-dipole-coupled states due to selection rules. These insights provide essential guidelines for the optimization of Boltzmann thermometers to operate in an extended temperature range.

## Abstract (web summary)

Boltzmann thermometers rely on the luminescence from energy levels in thermal equilibrium. Understanding of factors that enhance equilibration allows the design of lanthanide-based thermometers with extended operating range.

## Graphical abstract

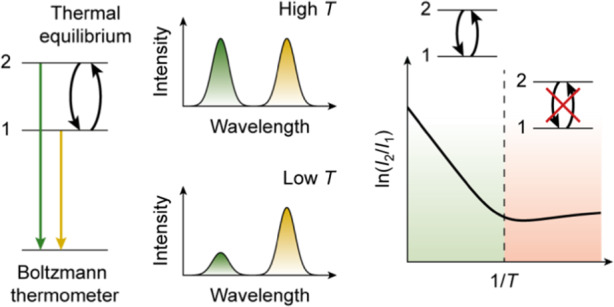

## Introduction

Over the last two decades, materials with temperature-sensitive luminescence have been developed as probes for remote thermometry. In particular, crystals doped with trivalent lanthanide (Ln^3+^) ions have received great interest. The luminescence of these ions is characterized by narrow emission lines that cover the deep ultraviolet until the near-infrared regions of the electromagnetic spectrum, making them suitable optical probes in various research fields^1–3^. The emission spectra of Ln^3+^ ions can strongly depend on temperature, which makes Ln^3+^-doped materials promising candidates for optical thermometry. The intensity ratio between two emission lines is most commonly used as measure for temperature because it is often insensitive to experimental factors that affect the integrated intensity such as alignment and the excitation intensity and it allows for the use of a simple measurement setup. How strongly the intensity ratio responds to temperature is governed by nonradiative transitions between excited states within the thermometer material.

An important class of ratiometric thermometers is based on two thermally coupled excited states within a single Ln^3+^ ion. Thermal coupling involves the interaction with one or multiple phonons to bridge the energy gap between the two states, which enables exchange of their populations. At elevated temperatures, these nonradiative transitions become faster than any other decay or feeding pathway and, as a result, the populations of the two states reach thermal equilibrium^4^. The well-known energy gap law dictates that excited states separated by smaller energy gaps reach thermal equilibrium at lower temperatures, because fewer phonons are required to bridge the gap resulting in faster nonradiative coupling. The luminescence intensity ratio (*LIR*) of two states in thermal equilibrium follows Boltzmann statistics, which serves as a reliable calibration model. The performance of Boltzmann thermometers is further quantified by the relative sensitivity $\Delta E/k_{{{\mathrm{B}}}}T^2$ and, in some cases, by the absolute sensitivity ${{{\mathrm{d}}}}LIR/{{{\mathrm{d}}}}T$. Suta and Meijerink used these parameters to propose an optimum temperature window *T* of $(0.3 - 0.5)$ $\Delta E/k_B$ for thermometry experiments based on the energy gap ∆*E*, assuming the two emitting states are in thermal equilibrium^4^. A stricter definition of the dynamic temperature range, in which Boltzmann thermometers have reliable performance, should additionally consider the onset temperature of thermal equilibrium. Especially at the lower temperature limit, Boltzmann equilibrium is not always realized. Insight into how the temperature window for Boltzmann equilibrium can be extended to cover the full optimum temperature window for high accuracy temperature sensing is important, but often ignored, the figure of merit for Boltzmann thermometers.

Recent studies have shown that at elevated dopant concentrations cross-relaxation between nearby thermometer ions can affect the establishment of thermal equilibrium. For instance, the ^4^F_3/2_ and ^4^F_5/2_ levels of Nd^3+^ experience strong cross-relaxation providing an additional depopulation path that competes with nonradiative coupling and thus causes an undesired increase of the onset temperature of thermal equilibrium^5^. Cross-relaxation from the ^5^D_1_ and ^5^D_0_ levels of Eu^3+^ lead to a desirable opposite effect^6^. Coupling with ^7^F_0_–^7^F_3_ and ^7^F_2_–^7^F_4_ transitions in nearby Eu^3+^ ions that are resonant with the ^5^D_1_–^5^D_0_ energy gap accelerates nonradiative coupling between the states, which shifts the onset of Boltzmann equilibrium to lower temperatures. However, only for specific Boltzmann thermometers can cross-relaxation extend the dynamic range and, so far, a positive impact is only reported for Eu^3+^. Universal methods to extend the dynamic temperature range of all Boltzmann thermometers are still lacking, which hampers their optimization for specific applications.

Here, we investigate how the host crystal and selection rules affect the dynamic range of Boltzmann thermometers. We prepare microcrystalline materials doped with low concentrations of Eu^3+^, serving as a model system for Boltzmann thermometry, and acquire the steady-state luminescence spectra and time-resolved luminescence from ^5^D_1_ and ^5^D_0_ to understand the impact of the host crystal on the nonradiative coupling rates between these levels. Our results show that the coupling rates increase with the maximum vibrational energy of the host crystal, which is in qualitative agreement with the energy-gap law. This reduces the onset of thermal equilibrium from 750 K for fluorides with maximum vibrational energies of 450 cm^−1^ to 450 K for complex oxides with 1200–1400 cm^−1^ vibrations. Secondly, we demonstrate that even within a series of isostructural fluorides it is possible to control the nonradiative rates via the lanthanide–fluoride distance and exploit the distance dependence of energy transfer between vibrational modes and the Eu^3+^ ion to shift the onset temperature by more than 100 K. Thirdly, we compare the onset temperature of Eu^3+^-doped materials with other Ln^3+^-based Boltzmann thermometers, which reveals an important role of selection rules: thermometers that rely on coupling between the emitting states by magnetic-dipole transitions, like Eu^3+^ and Nd^3+^, tend to have higher onset temperatures than thermometers with electric-dipolar nonradiative transitions. Finally, our results on Y_2_O_2_S:Eu^3+^ showed a surprising influence of additional nonradiative pathways on the thermometric performance of a luminescent Boltzmann thermometer. The new insights presented are essential for a better understanding of the performance of Boltzmann thermometers and for the design of thermometers with an optimized dynamic temperature range for specific applications.

## Results

**Fig. 1:**
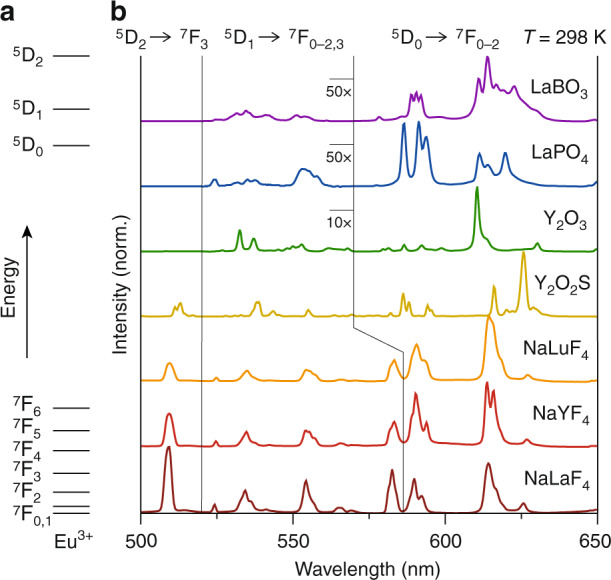
Eu^3+^ luminescence. **a** Energy level diagram of Eu^3+^. **b** Emission spectra of various Eu^3+^-doped host lattices acquired at room temperature. The luminescence of *β*-Na*RE*F_4_:Eu^3+^ (*RE* = La, Y, Lu) was excited at 395 nm into the ^5^L_6_ level, while Eu^3+^ in the other samples was excited into the ligand-to-metal charge-transfer band at 250–310 nm. To prevent cross-relaxation low Eu^3+^ concentrations of 0.4–0.5% were used in LaBO_3_, LaPO_4_, NaLuF_4_, NaYF_4_, and NaLaF_4_, while this required even lower dopant concentrations of 0.1% and 0.05% for Y_2_O_2_S and Y_2_O_3_, respectively. The vertical lines mark the wavelength range of the ^5^D_1_$\to$^7^F_0–3_ emissions of *β*-Na*RE*F_4_:Eu^3+^ and the ^5^D_1_$\to$^7^F_0–2_ emissions of the other materials. The emissions were magnified by a factor 10 for Y_2_O_3_ and by a factor of 50 for LaBO_3_ and LaPO_4_

We investigated the luminescence of Eu^3+^ in various host lattices to understand how the onset of thermal equilibrium between ^5^D_0_ and ^5^D_1_ can be controlled. The dopant concentration in each host (LaBO_3_, LaPO_4_, Y_2_O_3_, Y_2_O_2_S, Na*RE*F_4_ (*RE* = La, Y, Lu)) was below 0.5% to prevent an influence (lowering) of the onset temperature by cross-relaxation between Eu^3+^ ions. Figure 1b shows the luminescence spectra Eu^3+^ in all these materials acquired at room temperature. The spectra show characteristic Eu^3+^ luminescence with bright emission lines at 585–630 nm due to the ^5^D_0_→^7^F_1–2_ transitions^6^. The spectra show additional lines at 520–570 nm and 510 nm which are assigned to ^5^D_1_→^7^F_0–2_ and ^5^D_2_→^7^F_3_ transitions, respectively^3^. In the fluorides, we additionally observe relatively strong ^5^D_1_→^7^F_3_ emissions due to slow multi-phonon relaxation. A careful selection of the spectral integration range to separate the ^5^D_1_→^7^F_3_ and ^5^D_0_→^7^F_1_ emission is needed indicated by the vertical lines in Fig. 1b. The intensity of ^5^D_1_ and ^5^D_2_ emissions decreases as the maximum vibrational energy of the host lattice increases going from *β*-Na*RE*F_4_ (450 cm^−1^), Y_2_O_2_S (550 cm^−1^), Y_2_O_3_ (600 cm^−1^), LaPO_4_ (1200 cm^−1^), to LaBO_3_ (1400 cm^−1^)^7^. This indicates that nonradiative relaxation becomes faster when less phonons are required to bridge the gap to the first lower excited state, which is in agreement with the energy-gap law^8–10^. Comparing the spectra of the *β*-Na*RE*F_4_, which all have similar vibrational energies, the ^5^D_1_ and ^5^D_2_ emissions show an increasing trend in emission intensity going from a shorter (*β*-NaLuF_4_) to a longer (*β*-NaLaF_4_) lanthanide–ligand distance. This hints towards an inverse scaling of nonradiative coupling rates with the lanthanide–ligand distance, which is the expected trend for energy transfer to lattice vibrations^8,11^. Remarkably, the ^5^D_1_ and ^5^D_2_ emissions in Y_2_O_2_S are much stronger compared to Y_2_O_3_, while the vibrational energies are similar. A plausible explanation is the enhanced admixture of the low-energy Eu^3+^←S^2−^ charge-transfer state in Y_2_O_2_S with the energy levels of Eu^3+^, which increases the radiative rates for forced electric-dipole transitions from ^5^D_1_ and ^5^D_2_ but not nonradiative magnetic-dipole transitions between ^5^D_1_ and ^5^D_0_. In the following sections, we analyze the time- and spectrally resolved luminescence acquired at 7–873 K to further study these observations and, more importantly, determine the influence of the host lattice and selection rules on the onset of thermal equilibrium.

### The required number of phonons

**Fig. 2:**
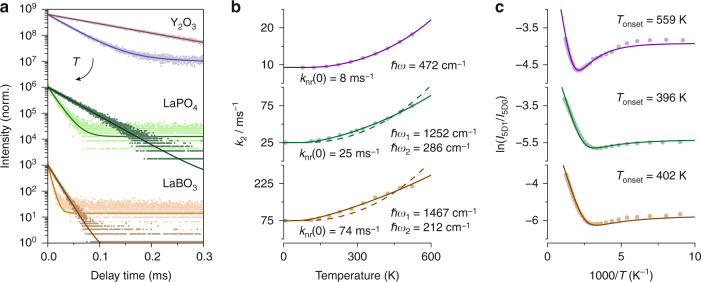
Relation between the maximum phonon energy and the onset of thermal equilibrium. **a** Luminescence decay curves of the ^5^D_1_ emission for Y_2_O_3_ (top), LaPO_4_ (middle), and LaBO_3_ (bottom). The luminescence was excited at ^7^F_0_ → ^5^D_1_ and the decay curves were measured at 78 K (dark color, slower decay) and 523 K (light color, faster decay). **b** Decay rates of the ^5^D_1_ emission at various temperatures. The solid line in the top plot and the dashed lines in the middle and bottom plots are fits of the experimental rates (dots) to Eq. 1, while the solid lines in the middle and bottom plots are fits to Eq. 3. **c** Intensity ratios of the ^5^D_1_ and ^5^D_0_ emissions for various temperatures determined from luminescence spectra upon excitation into the charge-transfer states. The solid lines are fits of the experimental ratios (dots) to the model of Eq. 4 for Y_2_O_3_ and Eq. 5 for LaBO_3_ and LaPO_4_ (lines). The onset temperatures for thermal equilibrium (*T*_onset_) were calculated using Eq. 6 for Y_2_O_3_:Eu^3+^ and a numerical solution was used for LaPO_4_:Eu^3+^ and LaBO_3_:Eu^3+^ (see text for further details)

First, we study the relation between the maximum phonon energy of the host lattice and the onset of thermal equilibrium, where we focus on Y_2_O_3_, LaPO_4_, and LaBO_3_. Figure 2a shows the luminescence decay curves of the ^5^D_1_ emission for these materials at various temperatures. We observe single-exponential decay at 78 K, which confirms that cross-relaxation is negligible as this would cause highly multi-exponential decay^6^. At elevated temperatures, decay from ^5^D_1_ becomes faster, because the rate of multi-phonon relaxation to ^5^D_0_ increases as phonon modes are thermally occupied. The measurements at elevated temperatures additionally show a slow component—in LaPO_4_ and LaBO_3_, this is barely distinguishable from the background signal due to the low amplitude of this component. Figure S1 shows decay curves for Y_2_O_3_:Eu^3+^ on an extended time scale, in which the slow component is clearly visible and which demonstrate that the decay rate of the slow component matches the population-weighted average radiative decay rate of the thermally coupled ^5^D_1_ and ^5^D_0_ levels, in agreement with the work of Geitenbeek et al*.*^6^. The fast component contains information on the temperature dependence of multi-phonon relaxation and is used to further analyze nonradiative coupling.

The decay rate of the fast component *k*_2_ is described as the sum of temperature-independent radiative decay with rate *k*_r,2_ and a temperature-dependent multi-phonon relaxation term^4,12^

1 $$k_2 = k_{{{{\mathrm{r}}}},2} + k_{{{{\mathrm{nr}}}}}(0)g_1(1 + n)^p\;{{{\mathrm{with}}}}\;n = \frac{1}{{\exp \left( {\hbar \omega /k_{{{\mathrm{B}}}}T} \right) - 1}}$$

where *k*_nr_(0) is the intrinsic nonradiative rate, *g*_1_ is the degeneracy of the lower excited state, *p* is the number of phonons needed to bridge the gap, *n* is the phonon occupation number, and $\hbar \omega$ is the energy of the effective phonon mode involved. Multi-phonon relaxation is thus governed by spontaneous phonon emission that is independent of temperature and by stimulated phonon emission that increases with temperature as phonon modes are thermally occupied. We determine $k_{{{{\mathrm{r}}}},2}$ and $k_{{{{\mathrm{nr}}}}}(0)$ using luminescence measurements at low temperatures with negligible stimulated phonon emission. Specifically, we determine $k_2$ from the ^5^D_1_ decay curve using a single-exponential fit and then determine $k_{{{{\mathrm{nr}}}}}(0)$ and $k_{{{{\mathrm{r}}}},2}$ using the intensity ratio between the ^5^D_1_ and ^5^D_0_ emissions in the low-temperature luminescence spectrum excited at ^7^F_0_→^5^D_1_ (Sec. S1). This yields values for $k_{{{{\mathrm{nr}}}}}(0)$ of 8, 25, and 74 ms^−1^ for Y_2_O_3_, LaPO_4_, and LaBO_3_, respectively, which shows an increasing trend with the maximum vibrational energy of the host. This is in agreement with the energy-gap law, formulated by van Dijk and Schuurmans^8–10^

2 $$k_{{{{\mathrm{nr}}}}}\left(0 \right) = A\exp \left({ - \gamma p} \right)$$

where constants *A* and *γ* depend on the host lattice and on the dipole moment of the nonradiative coupling transition. This indicates that host lattices with higher vibrational energies have faster nonradiative coupling, which can lower the onset temperatures of thermal equilibrium. The energy-gap law as discussed by Van Dijk and Schuurmans also shows that the nonradiative rates scale with the (electric-)dipole transition probability between the two levels.

Next, we determine the rates of the fast component from ^5^D_1_ decay curves recorded at various temperatures (Fig. 2b). At low temperatures, we used a single-exponential fit, while a bi-exponential fit was necessary to match the experiments at elevated temperatures. The decay rates increase with temperature, as we observed before in Fig. 2a. We first analyze this trend for Y_2_O_3_ by fitting the experimental rates (dots) to Eq. 1 (solid line), where we optimized the phonon energy $\hbar \omega$ and order *p* of the phonon process while keeping $k_{{{{\mathrm{nr}}}}}(0)$ and $k_{{{{\mathrm{r}}}},2}$ fixed. For all Eu^3+^-based thermometers of this study, we fixed the energy gap between ^5^D_1_ and ^5^D_0_ to 1750 cm^−1^, corresponding to the typical reported value. The fit is in excellent agreement with the experiment and yields values of $\hbar \omega =$ 472 cm^−1^ and *p* = 3.7. In this case, multi-phonon relaxation is best described by a non-integer number of phonons, which indicates that the process is in reality more complex than described by Eq. 1— for instance, multiple phonon modes with slightly different energy can participate in the nonradiative transition. Where in the simple multi-phonon relaxation picture a vibrational overtone of a single phonon mode is considered to bridge the energy gap, in reality multiple combinations of vibrational modes with different energies can be used to bridge the gap in parallel processes. It is impossible to capture and model all these different combinations of vibrational modes that contribute to multi-phonon relaxation. We, therefore, approximate that nonradiative transitions take place via an effective non-integer number of phonons.

To analyze the ^5^D_1_ decay rates of Eu^3+^-doped LaPO_4_ and LaBO_3_, we again fit the experimental data (dots) to Eq. 1 (dashed lines). In this case, the model poorly matches the experiment, so multi-phonon relaxation likely involves more than one effective phonon mode. We, therefore, adjust the model to allow one phonon with higher-energy $\hbar \omega _1$ and occupation $n_1$ and a non-integer number of phonons $p_2$ with lower energy $\hbar \omega _2$ and occupation $n_2$:

3 $$k_2 = k_{{{{\mathrm{r}}}},2} + k_{{{{\mathrm{nr}}}}}(0)g_1(1 + n_1)(1 + n_2)^{p_2}$$

We fit the experimental decay rates to Eq. 3 and find for both LaPO_4_ and LaBO_3_ an excellent match between model and experiment. For LaPO_4_, we find that the transition takes place via one phonon of 1252 cm^−1^ and 1.7 phonons of 286 cm^−1^, while in LaBO_3_ one phonon of 1467 cm^−1^ and 1.3 phonons of 212 cm^−1^ give the best fit. At temperatures relevant for thermometry (<1000 K), the occupation of these high-energy modes is negligible compared to the occupation of the low-energy modes. The high-energy modes that participate in the transition are thus spontaneously emitted, while the temperature dependence is determined by the increasing population of the low-energy modes.

To monitor and understand the onset temperature for thermal equilibrium we study the luminescence intensity ratio between the ^5^D_1_ and ^5^D_0_ emissions, which is the relevant metric for Eu^3+^-based thermometry. Figure 2c shows the ^5^D_1_/^5^D_0_ ratios for various temperatures, which presents a similar trend for the three studied materials. At low temperatures, the ratio remains constant when the thermal occupation of phonon modes is still negligible. At higher temperatures, the phonon occupation increases, which initially enhances relaxation from ^5^D_1_ to ^5^D_0_ via stimulated phonon emission, and later also boosts excitation from ^5^D_0_ to ^5^D_1_ via (stimulated) phonon absorption—the latter depends on temperature as $n(T)^p$. In the region between these regimes, we observe a minimum in the intensity ratios, because the phonon emission rate increases faster with temperature than the phonon absorption rate (Sec. S2). At temperatures beyond the minimum, the intensity ratio increases, and thermal equilibrium is established, which causes the typical Boltzmann behavior: a linear relation between the logarithm of intensity ratio and reciprocal temperature.

To further understand the observations in Fig. 2c we consider the analytical intensity ratio of two excited states that can radiatively decay and are thermally coupled via phonon emission and absorption pathways. The system is excited into a higher-energy auxiliary state, from which feeding to the two thermally coupled levels takes place. Assuming that one effective phonon mode participates in the coupling pathway, the steady-state solution of the rate equations gives the following expression for the intensity ratio^4,6^

4 $$\frac{{I_2}}{{I_1}} = C\frac{{k_{{{{\mathrm{r}}}},1}\alpha + k_{{{{\mathrm{nr}}}}}(0)g_2n^p}}{{k_{{{{\mathrm{r}}}},2}(1 - \alpha ) + k_{{{{\mathrm{nr}}}}}(0)g_1(1 + n)^p}}$$

Where $k_{{{{\mathrm{r}}}},1}$ and $k_{{{{\mathrm{r}}}},2}$ are the radiative decay rates from the lower (^5^D_0_) and higher (^5^D_1_) thermally coupled levels, respectively, and pre-factor *C* is the ratio between the Einstein coefficients for spontaneous photon emission from these states ($A_2$/$A_1$) to lower states involved in the determination of intensity ratio ($I_2$/$I_1$). Feeding factor *α* gives the fraction of the auxiliary-state population that feeds directly into the higher thermally coupled state, while the remaining part $(1 - \alpha )$ populates the lower thermally coupled state. In the three oxide host lattices, feeding is dominated by multi-phonon relaxation indicated by the absence of ^5^D_2_ emissions, which sets *α* to 1. We fit the experimental ratios of Y_2_O_3_:Eu^3+^ to Eq. 4, where we separately determined $k_{{{{\mathrm{r}}}},1}$ from a decay curve of ^5^D_0_ at 7 K, leaving *C* as the only fitting parameter. For LaPO_4_:Eu^3+^ and LaBO_3_:Eu^3+^, we need a modified expression for the intensity ratio to account for the participation of two different phonon modes in the thermal coupling transition

5 $$\frac{{I_2}}{{I_1}} = C\frac{{k_{{{{\mathrm{r}}}},1}\alpha + k_{{{{\mathrm{nr}}}}}(0)g_2n_1n_2^{p_2}}}{{k_{{{{\mathrm{r}}}},2}(1 - \alpha ) + k_{{{{\mathrm{nr}}}}}(0)g_1(1 + n_1)(1 + n_2)^{p_2}}}$$

We fit the experimental intensity ratios of LaPO_4_:Eu^3+^ and LaBO_3_:Eu^3+^ to Eq. 5 with again *C* as the only fitting parameter. For all three materials, we find an excellent agreement between model and experiment, which confirms that, in Eu^3+^-doped Y_2_O_3_, LaPO_4_, and LaBO_3_, thermal coupling determines the temperature dependence of the intensity ratio between ^5^D_1_ and ^5^D_0_.

To compare the dynamic range of these thermometer materials we determine the onset of thermal equilibrium. In the absence of additional nonradiative transitions like cross-relaxation, thermal equilibrium starts when phonon emission and absorption become faster than radiative decay. Since phonon absorption is a purely stimulated process and contains no spontaneous contribution, its rate is always lower than the phonon emission rate. Assuming that the radiative rates of the thermally coupled levels are of the same order of magnitude, thermal equilibrium is therefore limited by the competition between phonon absorption and radiative decay within the lower level. If one effective phonon mode is involved, we can thus derive the expected onset temperature from the condition $k_{{{{\mathrm{r}}}},1} = k_{{{{\mathrm{nr}}}}}(0)g_2n^p$:

6 $$T_{{{{\mathrm{onset}}}}} = \frac{{\Delta E/p}}{{k_{{{\mathrm{B}}}}{{{\mathrm{ln}}}}\left[ {1 + \left( {\frac{{g_2k_{{{{\mathrm{nr}}}}}(0)}}{{k_{{{{\mathrm{r}}}},1}}}} \right)^{1/p}} \right]}}$$

We find an onset temperature of 559 K for Eu^3+^ in Y_2_O_3_. The above condition changes to $k_{{{{\mathrm{r}}}},1} = k_{{{{\mathrm{nr}}}}}(0)g_2n_1n_2^{p_2}$ for LaPO_4_:Eu^3+^ and LaBO_3_:Eu^3+^, which has no simple solution for $T_{{{{\mathrm{onset}}}}}$ but can be solved numerically, giving onset temperatures of 396 K and 402 K, respectively. Clearly, the onset temperature is lower in host lattices with higher vibrational energies, which is thus an important parameter to control and optimize the dynamic range of Boltzmann thermometers.

### The lanthanide–ligand distance

**Fig. 3:**
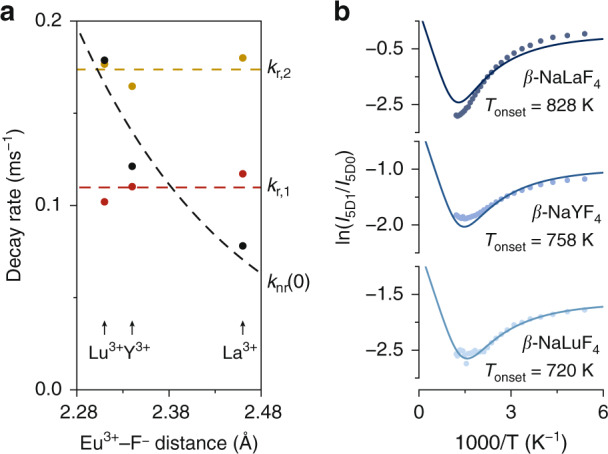
Tuning the intrinsic nonradiative rate via the lanthanide–ligand distance. **a** Radiative rates of ^5^D_0_ ($k_{{{{\mathrm{r}}}},1}$) and ^5^D_1_ ($k_{{{{\mathrm{r}}}},2}$) and the intrinsic nonradiative rate between these levels $k_{{{{\mathrm{nr}}}}}(0)$ for Eu^3+^ in *β*-NaLuF_4_, *β*-NaYF_4_, and *β*-NaLaF_4_. Similar to the procedure described in Sec. S1, luminescence measurements at 7 K were used to determine these rates. The dashed lines serve as guide to the eye. **b** Intensity ratios between the ^5^D_1_ and ^5^D_0_ emissions for various temperatures determined from luminescence spectra upon excitation at 395 nm in ^5^L_6_. The solid lines are the result of a global fit of the experimental ratios (dots) to the model of Eq. 4 (lines), where one value of pre-factor $C$ and one value of the vibrational energy $\hbar \omega$ and three different values of feeding factor $\alpha$ were used as fitting parameters. The onset temperatures were calculated using Eq. 6

We further investigate the impact of the lanthanide–ligand distance on the nonradiative transitions using Eu^3+^-doped *β*-NaLuF_4_, *β*-NaYF_4_, and *β*-NaLaF_4_. In these materials, the Eu^3+^–F^−^ distance varies without a change in their hexagonal crystal structure^13,14^. We use crystal structure data (acquired at room temperature) to determine the average Eu^3+^–F^−^ distances in *β*-NaLuF_4_ (2.31 Å), *β*-NaYF_4_ (2.34 Å), and *β*-NaLaF_4_ (2.46 Å). Similar to the procedure described in Sec. S1, we record the luminescence of these materials at 7 K and determine $k_{{{{\mathrm{nr}}}}}(0)$ and $k_{{{{\mathrm{r}}}},2}$ using the decay curve of the ^5^D_1_ emission and the ^5^D_1_/^5^D_0_ intensity ratio in the luminescence spectrum excited at ^7^F_0_→^5^D_1_ (Fig. 3a). In addition, we extract $k_{{{{\mathrm{r}}}},1}$ from the decay curve of the ^5^D_0_ emission. The analysis clearly reveals that the radiative rates are insensitive to the Eu^3+^–F^−^ distances in this series of isostructural host lattices. This is also expected as the Eu^3+^ ions share the same local site symmetries in *β*-Na*RE*F_4_ and selection rules therefore have similar impact although slight variations in the crystal field strength could explain small differences in the radiative rates. In contrast, the intrinsic nonradiative rate $k_{{{{\mathrm{nr}}}}}(0)$ strongly decreases with increasing Eu^3+^–F^−^ distance from 0.18 ms^−1^ for *β*-NaLuF_4_ to 0.08 ms^−1^ for *β*-NaLaF_4_. Ermolaev and Sveshnikova observed a qualitatively similar distance dependence for coupling of excited lanthanide and transition-metal ions to solvent vibrations, which they interpreted as dipole–dipole energy transfer between the electronic transition dipole moment and the dipole moment of the vibrational mode^8,11^. If the transitions involved are two localized electric dipoles with a distance between the dipoles that are much larger than their spatial extension, the nonradiative rate can be described as Förster-type energy-transfer that scales inversely with distance to the sixth power. This is a valid approximation for coupling with distant solvent vibrations, which was demonstrated by the analysis of solvent quenching in Er^3+^-doped NaYF_4_ nanocrystals^15^. However, the nonradiative transitions between ^5^D_1_ and ^5^D_0_ have a dominant magnetic-dipole character and the lattice vibrations spatially overlap with the transition of the Eu^3+^ ion. The strong variation in $k_{{{{\mathrm{nr}}}}}(0)$ explains the large decrease in ^5^D_1_ emission intensity from NaLaF_4_:Eu^3+^ to NaLuF_4_:Eu^3+^. The 2.3 times increase (0.08 ms^−1^ to 0.18 ms^−1^ from NaLaF_4_ to NaLuF_4_) is even stronger than a 1.5 times increase that is expected based on the $R^{ - 6}$ distance dependence of Förster-type energy transfer. The decreasing trend of the intrinsic nonradiative rate with increasing lanthanide–ligand distance is evident and may be qualitatively understood as follows: the oscillating charge density due the surrounding lattice motion induces an electromagnetic field, which has a gradually weaker amplitude at the position of the Eu^3+^ ions for larger Eu^3+^–ligand distances. However, further work is required to quantitively understand the observed distance dependence in a regime where the point dipole approximation is no longer valid.

Figure 3b presents the ^5^D_1_/^5^D_0_ intensity ratios of the three Eu^3+^-doped fluorides at various temperatures and shows a similar trend as observed in the oxides (Fig. 2c). In Fig. 1, we noted intense ^5^D_2_ emissions in the luminescence spectra of the fluorides acquired at room temperature. A long(er) lived ^5^D_2_ state does not exclusively relax to the ^5^D_1_ state by multi-phonon relaxation but allows for substantial radiative feeding from ^5^D_2_ to both ^5^D_1_ and ^5^D_0_ and, thus, no longer allows us to set *α* to 1^16^. In addition, we can assume that nonradiative coupling in the three *β*-NaREF_4_ hosts takes place via one effective vibrational mode with equal energy, since the effective mass of the Eu^3+^–F^−^ units is the same^14^. Furthermore, Fig. 3a demonstrates that the radiative rates of Eu^3+^ in the *β*-Na*RE*F_4_ hosts are very similar and we thus expect that one value of pre-factor *C* describes all intensity ratios. We, therefore, perform a global fit on the experimental ratios of all three materials to Eq. 4 to find one value of $\hbar \omega$ and one value of *C*, where we use three different feeding factors *α* as additional fitting parameters and the values of $k_{{{{\mathrm{r}}}},1}$ and $k_{{{{\mathrm{nr}}}}}(0)$ from Fig. 3a as input parameters. This yields a value for $\hbar \omega$ of 414 cm^−1^ and values for *α* of 0.76, 0.75, and 0.83 for *β*-NaLuF_4_, *β*-NaYF_4_, and *β*-NaLaF_4_, respectively, showing no clear trend with Eu^3+^–F^−^ distance. However, we would expect a decrease of *α* with increasing distance due to reduced multi-phonon relaxation rates and, therefore, a longer-lived ^5^D_2_ state with a stronger contribution of radiative feeding to ^5^D_0_ bypassing the ^5^D_1_ state. The absence of this trend and the poor quality of the fits indicate that the description of feeding by a constant *α* is not completely correct. Instead, *α* should likely depend on temperature, since feeding of the ^5^D_1_ state by multi-phonon relaxation is also temperature-dependent. Designing a model that includes the temperature dependence of feeding is complex and beyond the scope of this study. Nevertheless, we can still determine an approximate onset temperature using the obtained $k_{{{{\mathrm{nr}}}}}(0)$, $k_{{{{\mathrm{r}}}},1}$, and $\hbar \omega$, which are reliable and yield values for $T_{{{{\mathrm{onset}}}}}$ of 720 K, 758 K, and 828 K for *β*-NaLuF_4_, *β*-NaYF_4_, and *β*-NaLaF_4_, respectively. As expected from the lower $k_{{{{\mathrm{nr}}}}}(0)$ and $\hbar \omega$, the onset temperatures in the fluorides are much higher than in the oxides. Moreover, these results demonstrate that reducing the Eu^3+^–F^−^ distance by only 0.15 Å lowers the onset temperature by more than 100 K, which confirms the importance of the lanthanide–ligand distance in controlling the dynamic range of Boltzmann thermometers.

### Selection rules

So far, we have demonstrated how the host lattice affects nonradiative coupling for one specific transition, but a more generalized insight requires comparison of different thermometer ions and different pairs of thermally coupled levels. Besides the ^5^D_0_–^5^D_1_ pair in Eu^3+^, other popular Boltzmann thermometers use the ^4^F_3/2_–^4^F_5/2_ pair in Nd^3+^ ($\Delta E \approx$ 1000 cm^−1^), the ^6^P_7/2_–^6^P_5/2_ pair in Gd^3+^ ($\Delta E \approx$ 600 cm^−1^), the ^3^P_0_–^3^P_1_ pair in Pr^3+^ ($\Delta E \approx$ 600 cm^−1^), the ^4^S_3/2_–^2^H_11/2_ pair in Er^3+^ ($\Delta E \approx$ 700 cm^−1^), and the ^4^F_9/2_–^4^I_15/2_ pair in Dy^3+^ ($\Delta E \approx$ 1000 cm^−1^). One may expect relatively low nonradiative coupling rates between the emitting states in Eu^3+^, Nd^3+^, Gd^3+^, and Pr^3+^, because the transitions have dominant magnetic-dipole character as $\Delta J =$ 1 and the reduced matrix elements 〈||*U*^(2)^||〉^2^, 〈||*U*^(4)^||〉^2^, and 〈||*U*^(6)^||〉^2^ describing electric-dipole transitions are small for these transitions. In those cases, the reduced matrix element 〈||**L**+*g*_S_**S**||〉^2^ describing magnetic dipolar transitions (with **L** as orbital and **S** as spin angular momentum) are relatively large^16^. In contrast, selection rules predict high coupling rates in Er^3+^- and Dy^3+^-based thermometers that rely on electric-dipole transitions as is evident from the large 〈||*U*^(2)^||〉^2^, 〈||*U*^(4)^||〉^2^, and 〈||*U*^(6)^||〉^2^ values for the transition between the ^4^S_3/2_–^2^H_11/2_ levels in Er^3+^, and the ^4^F_9/2_–^4^I_15/2_ levels in Dy^3+^. For thermometers with similar energy gaps, it was previously demonstrated that the nonradiative rates of magnetic-dipole transitions are typically two to three orders of magnitude lower than of electric-dipole transitions^4^.

**Fig. 4:**
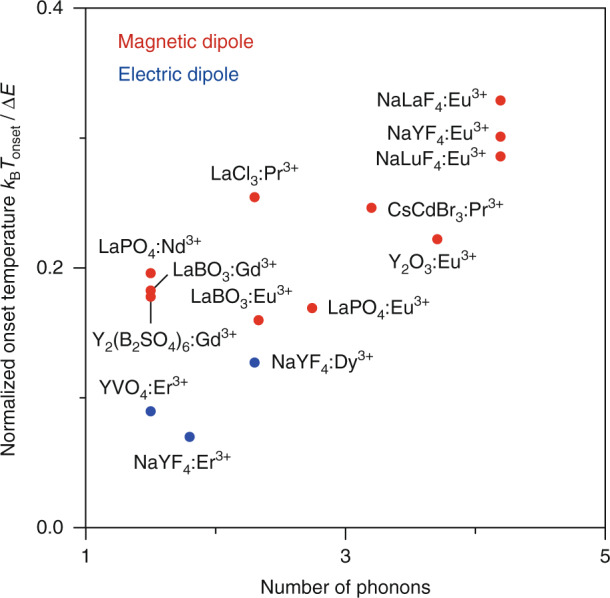
Normalized onset temperatures for various Boltzmann thermometers. All onset temperatures were calculated using Eq. 6 and normalized to $\Delta E/k_{{{\mathrm{B}}}}$. The results of Figs. 2 and 3 were used to calculate the onset temperatures of the Eu^3+^-based thermometers. Decay rates were extracted from literature to calculate the onset temperatures of CsCdBr_3_:Pr^3+^ ($k_{{{{\mathrm{nr}}}}}\left( 0 \right) =$ 1.5 × 10^2^ ms^−1^, $k_{1,{{{\mathrm{r}}}}} =$ 51 ms^−1^)^27^, LaCl_3_:Pr^3+^ ($k_{{{{\mathrm{nr}}}}}\left( 0 \right) =$ 3.1 × 10^2^ ms^−1^, $k_{1,{{{\mathrm{r}}}}} =$ 68 ms^−1^)^28,29^, LaPO_4_:Nd^3+^ ($k_{{{{\mathrm{nr}}}}}\left( 0 \right) =$ 57 ms^−1^, $k_{1,{{{\mathrm{r}}}}} =$ 2.3 ms^−1^)^5^, LaBO_3_:Gd^3+^ ($k_{{{{\mathrm{nr}}}}}\left( 0 \right) =$ 11 ms^−1^, $k_{1,{{{\mathrm{r}}}}} =$ 0.29 ms^−1^)^30^, Y_2_(B_2_SO_4_)_6_:Gd^3+^ ($k_{{{{\mathrm{nr}}}}}\left( 0 \right) =$ 8.9 ms^−1^, $k_{1,{{{\mathrm{r}}}}} =$ 0.21 ms^−1^)^30^, YVO_4_:Er^3+^ ($k_{{{{\mathrm{nr}}}}}\left( 0 \right) =$ 3.0 × 10^4^ ms^−1^, $k_{1,{{{\mathrm{r}}}}} =$ 5.1 ms^−1^)^31^. Decay rates were measured to calculate the onset temperatures of NaYF_4_:Er^3+^ ($k_{{{{\mathrm{nr}}}}}\left( 0 \right) =$ 1.9 × 10^5 ^ms^−1^, $k_{1,{{{\mathrm{r}}}}} =$ 1.4 ms^−1^) and NaYF_4_:Dy^3+^ ($k_{{{{\mathrm{nr}}}}}\left( 0 \right) =$ 2.0 × 10^2^ ms^−1^, $k_{1,{{{\mathrm{r}}}}} =$ 1.3 ms^−1^). In principle, the energy gap of LaPO_4_:Nd^3+^, LaBO_3_:Gd^3+^, Y_2_(B_2_SO_4_)_6_:Gd^3+^, and YVO_4_:Er^3+^ could be bridged by one phonon mode, but Fig. 2b suggested that more than one mode is typically necessary to realize resonance when the gap is small compared to the phonon energy. We, therefore, determine the onset temperature by inserting both 1 and 2 phonons in Eq. 6 and using the average $p$ and $T_{{{{\mathrm{onset}}}}}$

To understand how selection rules affect the onset of thermal equilibrium we extract $k_{{{{\mathrm{nr}}}}}(0)$, $k_{{{{\mathrm{r}}}},1}$, $\Delta E$, and $p$ from studies on various Boltzmann thermometers and determine the onset temperature using Eq. 6. These parameters are available in literature for multiple thermometers with magnetic-dipole transitions, but only for one thermometer with electric-dipole transitions (YVO_4_:Er^3+^, $k_{{{{\mathrm{nr}}}}}\left( 0 \right) =$ 3.0 × 10^4 ^ms^−1^). To ensure reliable comparison we measured the luminescence of two additional thermometers with electric-dipole transitions: *β*-NaYF_4_:Er^3+^ and *β*-NaYF_4_:Dy^3+^. At cryogenic temperatures, nonradiative decay from ^2^H_11/2_ in Er^3+^ and ^4^I_15/2_ in Dy^3+^ is already much faster than radiative decay, allowing extraction of $k_{{{{\mathrm{nr}}}}}(0)$ directly from the corresponding decay curves (Table S1). We find $k_{{{{\mathrm{nr}}}}}(0)$ values of 1.9 × 10^5 ^ms^−1^ and 2.0 × 10^2 ^ms^−1^ and onset temperatures of 75 K and 174 K for Er^3+^ and Dy^3+^, respectively. Compared to *β*-NaYF_4_:Eu^3+^, the obtained value of $k_{{{{\mathrm{nr}}}}}(0)$ for *β*-NaYF_4_:Dy^3+^ is indeed three orders of magnitude higher. Interestingly, the value of $k_{{{{\mathrm{nr}}}}}(0)$ for *β*-NaYF_4_:Er^3+^ is higher than expected. We speculate that in addition to a large effect of selection rules also stronger electron–phonon coupling may contribute to the high $k_{{{{\mathrm{nr}}}}}(0)$ value in Er^3+^^17^. To enable fair comparison of different thermometers we normalized the obtained onset temperatures to $\Delta E/k_{{{\mathrm{B}}}}$. Figure 4 reveals that the normalized onset temperature generally increases with $p$ as expected from the energy-gap law. More importantly, the normalized onset temperature of thermometers with magnetic-dipole transitions is much higher than for thermometers with electric-dipole transitions. For a specific energy gap, experiments at low temperatures thus benefit from thermometers with electric-dipole transitions.

### An additional quenching pathway

**Fig. 5:**
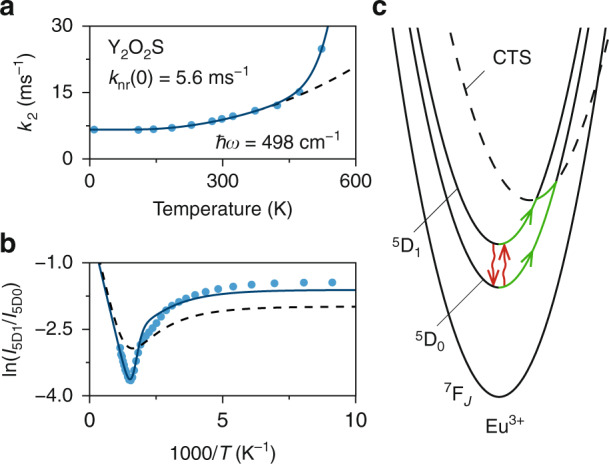
The impact of charge-transfer quenching on the onset of thermal equilibrium. **a** Decay rates of the ^5^D_1_ emission at various temperatures. The dashed black line is a fit of the experimental rates (blue dots) up to 423 K to Eq. 1, while the solid blue line is a fit to Eq. 7. **b** Intensity ratios between the ^5^D_1_ and ^5^D_0_ emissions for various temperatures extracted from luminescence spectra. The dashed black line is a fit of the experimental ratios (blue dots) to Eq. 4, while the solid blue line is a fit of Eq. 8. **c** Configurational coordinate diagram of the ^5^D_0–1_ and ^7^F_*J*_ levels of Eu^3+^ including the charge-transfer state (CTS) of Y_2_O_2_S. Coupling between the ^5^D_1_ and ^5^D_0_ levels takes place directly via phonon emission and absorption (red arrows) and indirectly via crossover to the CTS (green arrows)

Besides modification of thermal coupling, the host lattice can also introduce additional nonradiative relaxation pathways via a higher excited state. Mostly, this is studied in relation to luminescence quenching caused by, for instance, thermally activated crossover^18^. To investigate how this affects thermal equilibrium we acquired the temperature-dependent luminescence of Eu^3+^ in Y_2_O_2_S, which has a low-lying S^2−^-to-Eu^3+^ charge-transfer state (CTS) due to the soft, polarizable nature of the S^2−^ ligands^19,20^. The temperature-dependent behavior is remarkably different from the other studied materials. This is already demonstrated by the decay rate of the ^5^D_1_ emission, which above 423 K drastically increase, more than expected for multi-phonon relaxation, with temperature (Fig. 5a). We attribute this to thermally activated crossover from the ^5^D_1_ level to the CTS of the host^18^. We account for crossover to the CTS by the addition of a Mott-Seitz term to the total decay rate of ^5^D_1_:

7 $$k_2 = k_{{{{\mathrm{r}}}},2} + k_{{{{\mathrm{CT}}}}}\exp \left( { - E_{{{{\mathrm{a}}}},2}/k_{{{\mathrm{B}}}}T} \right) + k_{{{{\mathrm{nr}}}}}(0)g_1(1 + n)^p$$

where $E_{{{{\mathrm{a}}}},2}$ is the activation barrier for crossover from ^5^D_1_ to the CTS and $k_{{{{\mathrm{CT}}}}}$ is the rate constant^21,22^. We again acquire the luminescence at 7 K and find an intrinsic nonradiative rate $k_{{{{\mathrm{nr}}}}}(0)$ of 5.6 ms^−1^ that is similar to Y_2_O_3_ as expected from the comparable vibrational energies^7^. In contrast, the obtained radiative rates $k_{{{{\mathrm{r}}}},1}$ of 2.3 ms^−1^ and $k_{{{{\mathrm{r}}}},2}$ of 1.0 ms^−1^ are relatively high, which is explained by mixing of the CTS into the ^5^D_*J*_ states. Admixture of opposite-parity states into 4f^*n*^ states induces forced electric-dipole transitions and is strongly enhanced if the energy difference is reduced, resulting in large Judd-Ofelt parameters *Ω*_2_, *Ω*_4_ and *Ω*_6_^23^. This can explain the high radiative decay rates. Note that a low-energy opposite-parity state can also enhance the nonradiative coupling rates if the transition has electric-dipole character. However, no enhancement is expected for the ^5^D_1_–^5^D_0_ transitions due to the magnetic-dipole nature of this transition. Using $k_{{{{\mathrm{nr}}}}}(0)$ and $k_{{{{\mathrm{r}}}},2}$ as input, we fit the experimental decay rates up to 423 K to Eq. 1 and obtain a $\hbar \omega$ value of 498 cm^−1^ (black dashed line). Then, we fit the full range of decay rates to Eq. 7 with the obtained value for $\hbar \omega$ and the reported value for $E_{{{{\mathrm{a}}}},2}$ (6100 cm^−1^) as additional input to find $k_{{{{\mathrm{CT}}}}}$ (1.6 × 10^8 ^ms^−1^)^19^. The excellent agreement between the data and the model (solid blue line) indicates that thermally activated crossover to the CTS is an additional nonradiative path from ^5^D_1_ for Eu^3+^ in Y_2_O_2_S.

Figure 5b shows the temperature-dependent intensity ratios of Y_2_O_2_S:Eu^3+^, in which we observe a much sharper minimum than in the other studied materials. Similar to the fluorides, we also observed strong ^5^D_2_ emissions in the luminescence spectra of Y_2_O_2_S:Eu^3+^ (Fig. 1), indicating substantial feeding via radiative decay. We fit the experimental ratios (blue dots) to Eq. 4 (black dashed line) with $\alpha$ and $C$ as free parameters. The poor quality of the fit demonstrates that thermal coupling in this material cannot be solely described by phonon emission and absorption. Instead, the CTS can introduce a second indirect nonradiative coupling pathway. Specifically, the CTS can be fed from ^5^D_1_ or ^5^D_0_, after which it relaxes back to one of these states (Fig. 5c). The steady-state solution to the rate equations of this extended system is

8 $$\frac{{I_2}}{{I_1}} = C\frac{{k_{{{{\mathrm{r}}}},1}\alpha + k_{{{{\mathrm{CT}}}}}\exp \left( { - E_{{{{\mathrm{a}}}},1}/k_{{{\mathrm{B}}}}T} \right) + k_{{{{\mathrm{nr}}}}}(0)g_2n^p}}{{k_{{{{\mathrm{r}}}},2}(1 - \alpha ) + k_{{{{\mathrm{CT}}}}}\exp \left( { - E_{{{{\mathrm{a}}}},2}/k_{{{\mathrm{B}}}}T} \right) + k_{{{{\mathrm{nr}}}}}(0)g_1(1 + n)^p}}$$

Here, $E_{{{{\mathrm{a}}}},1}$ is the activation barrier for crossover from ^5^D_0_, which we fix to $E_{{{{\mathrm{a}}}},2} + \Delta E$, leaving only $\alpha$ and $C$ as free parameters. A fit of the experimental ratios to this extended model (blue solid line) perfectly captures the sharp minimum, which confirms that indirect coupling via the CTS modifies the thermal equilibration between ^5^D_1_ and ^5^D_0_^19,24^. At lower temperatures, the quality of the fit decreases, which we again attribute to temperature dependence of the feeding term $\alpha$. Figure S2 shows that nonradiative coupling via the CTS dominates over phonon emission and absorption, which makes our definition of the onset temperature no longer valid. If we do determine the onset temperature using Eq. 6, it gives a value of 707 K. This is much higher than the onset in Y_2_O_3_, because the low-lying CTS causes faster radiative decay from ^5^D_0_. An additional disadvantage of the low-lying CTS is quenching of the luminescence via crossover to the ^7^F_*J*_ states, which reduces the brightness of the thermometer resulting in low measurement precisions^25^. These considerations imply that the CTS in Y_2_O_2_S:Eu^3+^ has a negative impact on the performance of Eu^3+^ as a luminescent thermometer performance.

## Discussion

The dynamic temperature range is one of the most important considerations for the selection of a thermometer for a specific application. For luminescent Boltzmann thermometers, there has been a strong focus on realizing high relative sensitivities, which can be controlled via the energy gap. The highest sensitivities are however found for thermometers with large energy gaps, but they inherently suffer from high onset temperatures of thermal equilibrium putting a lower limit on the dynamic temperature range. This is not always realized and can lead to deviations and systematic errors in the lower temperature regime if Boltzmann equilibrium is assumed but not yet established.

Our work highlights three methods to lower the onset temperature and extend the dynamic temperature range of Boltzmann thermometers by: (i) decreasing the number of required phonons to bridge the energy gap, (ii) reducing the lanthanide–ligand distances within the host, and (iii) selecting a thermometer with excited states coupled by electric-dipolar nonradiative transitions. All methods rely on maximizing the intrinsic coupling rates between the emitting states, which mainly determine the onset of thermal equilibrium. The lanthanide–ligand distance has the smallest, but still significant, effect on this rate, showing an increase of a factor two from *β*-NaLaF_4_ to *β*-NaLuF_4_ resulting in a reduction of $T_{{{{\mathrm{onset}}}}}$ by 100 K. In contrast, the intrinsic coupling rates increased by three orders of magnitude with a decrease in the average number of required phonons from 4.2 to 2.3. This caused a difference in the onset temperature of more than 400 K between *β*-NaLaF_4_:Eu^3+^ (823 K) and LaBO_3_:Eu^3+^ (402 K). We further observed that the intrinsic nonradiative rate is two to three orders of magnitudes higher in luminescent thermometers, in which the excited states are coupled by electric-dipole transitions compared to thermometers with magnetic-dipole transitions, indicating an important and so far underestimated role of selection rules.

We find the lowest onset temperatures for Er^3+^, as expected from the small energy gap of 700 cm^−1^ and the electric-dipole character of the nonradiative transitions. Er^3+^-based thermometers further benefit from a high oscillator strength of the ^4^I_15/2_ ↔ ^2^H_11/2_ transition, which leads to strong emission from the ^2^H_11/2_ state at relatively low thermal population and thus guarantees high measurement precisions^25^. An additional advantage of Er^3+^-based thermometers is the possibility of co-doping with Yb^3+^ to allow for efficient generation of upconversion luminescence. This makes Er^3+^ the preferred Boltzmann thermometer in many cases. Some specific applications however require thermometers with different emission energies or higher sensitivities at elevated temperatures. For instance, experiments in biological tissue are preferably performed with Nd^3+^ due to the high penetration depth of its infrared emissions, while the large energy gap of Eu^3+^ makes it the preferred thermometer for accurate measurements of elevated temperatures. However, the magnetic-dipole character of the nonradiative transitions and the large energy gap inherently restrict such experiments to elevated temperatures. The experiments and considerations discussed in this work can aid in the selection of the best host materials to improve the dynamic range of these ions for specific applications when no thermometers with electric-dipole transitions are available.

To summarize, we have experimentally demonstrated how the host lattice impacts nonradiative coupling between ^5^D_1_ and ^5^D_0_ in Eu^3+^-based thermometers and how it controls the onset temperature of thermal equilibrium. Higher vibrational energies and shorter lanthanide–ligand distances help to lower the onset temperature of thermal equilibrium. Comparing onset temperatures of thermometers based on different lanthanide ions revealed that selection rules modify the intrinsic nonradiative rate and result in wider dynamic ranges for thermometers with excited states coupled by electric-dipolar transitions. These findings not only offer a fundamental understanding of thermal equilibrium but also provide design rules for the rational optimization of Boltzmann thermometers.

## Materials and methods

### Chemicals

Sodium fluoride (98%, NaF), ammonium fluoride (99.8%, NH_4_F), yttrium fluoride (99.9%, YF_3_), yttrium nitrate hexahydrate (99.8%, Y(NO_3_)_3_·6H_2_O), lanthanum nitrate hexahydrate (99.999%, La(NO_3_)_3_·6H_2_O), and europium nitrate pentahydrate (99.9%, Eu(NO_3_)_3_·5H_2_O) were obtained from Sigma-Aldrich. Dysprosium fluoride (99.9%, DyF_3_) and europium fluoride (99.99%, EuF_3_) were purchased from Strem Chemicals. Lanthanum fluoride (99.99%, LaF_3_) and lutetium oxide (99.99%, Lu_2_O_3_) were acquired from ChemPUR. Ammonium oxalate monohydrate (99.7%, (NH_4_)_2_C_2_O_4_·H_2_O) was obtained from Baker chemicals. Ammonium phosphate monohydrate (99%, (NH_4_)_2_HPO_4_·H_2_O) was acquired from Merck. Boric acid (99.8%, H_3_BO_3_) was purchased from Merck. MilliQ water (H_2_O) was used for washing and the preparation of aqueous solutions.

### Synthesis

Cubic Y_2_O_3_:Eu^3+^(0.05%) was prepared via a co-precipitation procedure. Solutions of 0.9995 eq. of Y(NO_3_)_3_·6H_2_O and 0.0005 eq. of Eu(NO_3_)_3_·5H_2_O in 10 mL H_2_O and 3 eq. (NH_4_)_2_C_2_O_4_·H_2_O in 50 mL H_2_O were mixed to form a white precipitate, which was washed with H_2_O and placed in a drying oven at 373 K. The dried precipitate was heated in air at 1673 K for 8 hours.

Monoclinic LaPO_4_:Eu^3+^(0.5%) was prepared via a co-precipitation procedure. Solutions of 0.995 eq. of La(NO_3_)_3_·6H_2_O and 0.005 eq. of Eu(NO_3_)_3_·5H_2_O in 15 mL H_2_O and 1 eq. (NH_4_)_2_HPO_4_ in 15 mL H_2_O was mixed to form a white precipitate, which was washed with H_2_O and placed in a drying oven at 373 K. The dried precipitate was heated in air at 1273 K for 12 hours.

Orthorhombic LaBO_3_:Eu^3+^(0.5%) was prepared via a two-step procedure. First, La_2_O_3_:Eu^3+^(0.5%) was obtained by a co-precipitation procedure, similar to the synthesis of Y_2_O_3_:Eu^3+^(0.05%) but with 0.995 eq. of La(NO_3_)_3_·6H_2_O and 0.005 eq. of Eu(NO_3_)_3_·5H_2_O as reactants. The product was thoroughly mixed with 2 eq. of H_3_BO_3_ and heated in air at 1123 K for 12 hours.

Hexagonal $\beta$-NaLuF_4_:Eu^3+^(0.5%) was prepared via a solid-state reaction. First, LuF_3_ was synthesized by dissolution of Lu_2_O_3_ in concentrated hydrogen chloride and precipitation with a concentrated aqueous NH_4_F solution in a Teflon beaker. The precipitated raw fluoride was isolated, washed with H_2_O and ethanol, dried at 393 K, and crystallized at 973 K in a bed of NH_4_F. A mixture of 0.995 eq. of LuF_3_, 0.005 eq. of EuF_3_, and 1 eq. of NaBF_4_ was thoroughly ground and heated in N_2_ atmosphere at 648 K for 3 hours. Hexagonal $\beta$-NaLaF_4_:Eu^3+^(0.5%) was obtained using the same procedure but with 0.995 eq. of LaF_3_ and 2 eq. of NaBF_4_ as reactants and a heating step at 898 K for 6 hours.

Hexagonal $\beta$-NaYF_4_:Dy^3+^(0.4%) was prepared via a solid-state reaction, based on the work of Geitenbeek et al*.*^6^. A mixture of 0.996 eq. of YF_3_, 0.004 eq. of DyF_3_, 1 eq. of NaF, and 0.9 eq. of NH_4_F was thoroughly ground. The ground mixture was placed in the oven in N_2_ atmosphere with a flux of NH_4_F and it was heated at 573 K for 3 hours followed by a second heating step at 823 K for 8 hours.

Hexagonal $\beta$-NaYF_4_:Eu^3+^(0.4%)^6^, hexagonal Y_2_O_2_S: Eu^3+^(0.1%)^19^, and hexagonal $\beta$-NaYF_4_:Er^3+^(0.1%)^26^ were available from previous studies.

### Structural characterization and spectroscopic experiments

The crystal structure of the materials was confirmed with a Philips PW1700 X-ray powder diffractometer, used for NaREF_4_ and LaPO_4_, and a Malvern Panalytical Aeris Research diffractometer, used for Y_2_O_2_S, Y_2_O_3_, and LaBO_3_. Both instruments were equipped with a Cu K_*α*_ (*λ* = 1.5418 Å) radiation source. All materials were found to be phase pure (Fig. S3). The luminescence spectra, from which the data is shown in Figs. 1, 2c, 3b, and 5b, were recorded using an Ocean Optics QE Pro010451 CCD detector and a 450 W Xe lamp as excitation source. The white light from the Xe lamp was passed through the TMS300 double monochromator of an Edinburgh Instruments FLS920 spectrofluorometer to select the excitation wavelength. The luminescence spectra that were used to determine $k_{{{{\mathrm{nr}}}}}(0)$ and $k_{{{{\mathrm{r}}}},2}$ were acquired using a Triax 550 monochromator equipped with a Hamamatsu R928 photomultiplier tube and an Ekspla NT342B OPO laser (10 Hz) as the excitation source. The line width of the laser was 6 cm^−1^, which enabled highly selective excitation of the ^7^F_0_→^5^D_1_ transition. Luminescence decay measurements were performed using the same Triax monochromator and Ekspla laser, but with a Hamamatsu H7422 photomultiplier tube as a single-photon counting detector. The laser synchronization and detection signals were recorded with a PicoQuant Timeharp 260 time-correlated single-photon counting module. The temperature of the samples was controlled between 78 K and 873 K using a Linkam THMS600 heating stage. Measurements at 7 K were performed with an Oxford Instruments liquid-He cold-finger cryostat.

## Supplementary information

Supplemental material
