# Supplementary material for: Extending the dynamic temperature range of Boltzmann thermometers
Source: Light Sci Appl. 2022 Dec 8;11:343. doi: 10.1038/s41377-022-01028-8 (PMC9732288; doi:10.1038/s41377-022-01028-8)
Supplement: Supplementary file 1 — Supplemental material [file 41377_2022_1028_MOESM1_ESM.pdf]

Supplementary information for

Extending the dynamic temperature range of Boltzmann thermometers

T.P. van Swieten[1], J.M. Steenhoff[1], A. Vlasblom[1], R. de Berg[1], S.P. Mattern[1], F.T. Rabouw[1], M. Suta[1,2*], A. Meijerink[1*]

[1]Debye Institute for Nanomaterials Science, Utrecht University, Princetonplein 1, 3584 CC Utrecht, The Netherlands

[2]Inorganic Photoactive Materials, Heinrich Heine University Düsseldorf, Universitätsstraße 1, 40225 Düsseldorf, Germany

*Author to whom correspondence should be addressed; electronic mail:

markus.suta@hhu.de, a.meijerink@uu.nl

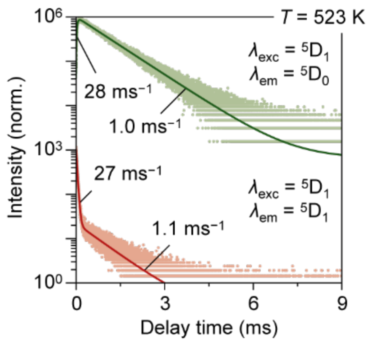

Figure S1. Luminescence decay of $Y_2O_3:Eu^{3+}$(0.05%) at 523 K. Decay curves of the $^5D_1$ and $^5D_0$ emission were recorded upon excitation into $^5D_1$. The experimental data was fitted to bi-exponential decay. Both the rates of the fast components and the rates of the slow components match, which is clear signature of thermal coupling (but not yet thermal equilibrium for which single exponential decay with the same decay time for $^5D_1$ and $^5D_0$ emission is observed).

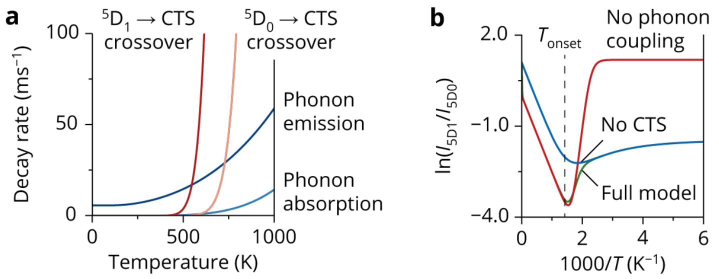

Figure S2. Nonradiative coupling between $^5D_1$ and $^5D_0$ in $Y_2O_2S:Eu^{3+}$. (a) Decay rates of crossover and phonon coupling from $^5D_1$ and $^5D_0$. (b) Calculated intensity ratios between $^5D_1$ and $^5D_0$ from Equation 8 of the main text (green), without phonon coupling terms (red), and without crossover rates.

Table S1. Experimental parameters of the thermally coupled $^5D_1$-$^5D_0$ levels of Eu^{3+} in different host lattices. The decay rates $k_{\mathrm{r},1}$, $k_{\mathrm{r},2}$, and $k_{\mathrm{nr}}(0)$ were obtained from luminescence measurements at 7 K or 78 K. Fitting procedures of the decay rates and intensity ratios to the analytical models in the main text yielded the energies of phonon modes, feeding factor α, pre-factor C, and rate constant k_{CT}. The onset of thermal equilibrium T_{onset} was calculated at the temperature, for which the phonon absorption rate is equal to the radiative rate from the lower thermally coupled state.

Material	$k_{r,1}$ (ms^{-1})	$k_{r,2}$ (ms^{-1})	$k_{nr}(0)$ (ms^{-1})	α	C	T_{onset} (K)	Phonon modes	Additional
$LaBO_3:Eu^{3+}$(0.5%)	0.98*	0.10	74	1.0	0.24	402	1×1467 cm^{-1}, 1.3×212 cm^{-1}	–
$LaPO_4:Eu^{3+}$(0.5%)	0.28	0.16	25	1.0	0.61	396	1×1252 cm^{-1}, 1.7×286 cm^{-1}	–
$Y_2O_3:Eu^{3+}$(0.05%)	0.98	0.50	8.0	1.0	0.16	559	3.7×472 cm^{-1}	–
$NaLuF_4:Eu^{3+}$(0.5%)	0.18	0.11	0.18	0.76	0.57	720	4.2×414 cm^{-1}	–
$NaYF_4:Eu^{3+}$(0.4%)	0.16	0.11	0.12	0.75	0.57	758	4.2×414 cm^{-1}	–
$NaLaF_4:Eu^{3+}$(0.5%)	0.18	0.10	0.08	0.83	0.57	828	4.2×414 cm^{-1}	–
$NaYF_4:Dy^{3+}$(0.4%)	1.3	–	2.0×10^{2}**	–	–	174	2.3×417 cm^{-1}	–
$NaYF_4:Er^{3+}$(0.1%)	1.4	–	1.9×10^{5}**	–	–	75	1.8×417 cm^{-1}	–
$Y_2O_2S:Eu^{3+}$(0.1%)	2.3	1.0	5.6	0.87	0.59	707	3.5×498 cm^{-1}	k_{CT} = 1.6×10^{8} ms^{-1}

* The decay curve of the $^5D_0$ emission is multi-exponential. We determined the average decay rate from a fit of the data to tri-expponential decay.

** Emission from the higher thermally coupled state was very weak, which made integration of the spectral line unreliable. $k_{nr}(0)$ was determined directly from the decay rate of this emission

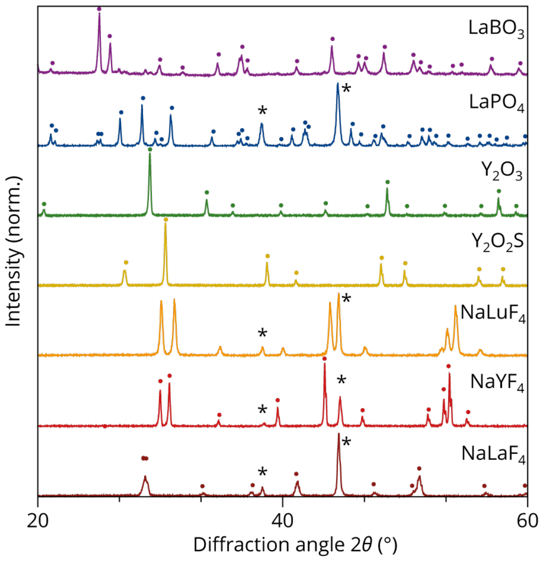

Figure S3. XRD patterns of the studied materials. The dots indicate the reflections that correspond to the crystal structure of each material. The reflections marked by an asterisk are due to the aluminum holder that was used to load the $NaREF_4$ and $LaPO_4$ samples. All materials are single phase and within the experimental detection limit of ~1% no other crystalline phases are present.

S1. Analysis of the temperature-dependent luminescence

We analyze the temperature dependence of the Eu^{3+} luminescence using decay curves and emission spectra. The goal of this analysis is to verify if the thermometer behaves as a system of two thermally coupled levels by fitting the experimental intensity ratios to Equation 4 or 5 of the main text. The number of unknown variables in Equation 4 and 5 is however too large to use all of them as fitting parameters. We therefore separately determine $k_{\mathrm{r},1}$, $k_{\mathrm{r},2}$, and $k_{\mathrm{nr}}(0)$ from luminescence measurements at 7 K, where the low temperatures suppress stimulated phonon emission and absorption or other undesired nonradiative processes. We use the decay rate of the $^5D_0$ emission as value for $k_{\mathrm{r},1}$, while the decay rate of the $^5D_1$ emission k_2 is the sum of $k_{\mathrm{r},2}$ and $k_{\mathrm{nr}}(0)$, as $g_1 = 1$. Since decay from $^5D_0$ is purely radiative, we can assume that at 7 K all $^5D_0$ emission upon excitation into $^5D_1$ is the sole result of spontaneous phonon emission from $^5D_1$. It is therefore possible to determine $k_{\mathrm{r},2}$ and $k_{\mathrm{nr}}(0)$ from k_2 using the emission spectrum covering all possible $^5D_{0\text{–}1}$ emissions upon excitation in $^7F_0 \longrightarrow {}^5D_1$:

$$k_{\mathrm{r},2} = \frac{k_2 I_2}{I_2 + I_1}$$

$$k_{\mathrm{nr}}(0) = \frac{k_2 I_1}{I_2 + I_1}$$

(S1)

Where I_2 and I_1 are the integrated intensities of the $^5D_1$ and $^5D_0$ emissions, respectively.

There are a few exceptions to this procedure. For Y_2O_3, we record the decay curve of the emission $^5D_1$ and the full-range spectrum at 78 K, because in this case the $^5D_1$ decay rate was slightly higher than the rate at 7 K, possibly due to differences in the decay rates of the crystal-field levels within $^5D_1$. For

$LaBO_3$, the decay curve of $^5D_0$ is multi-exponential at both 7 K and 78 K. We fit the decay curve measured at 78 K to three exponentials and used the average decay rate as $k_{\mathrm{r},1}$.

S2. The minimum in the temperature dependence of the intensity ratios

Assuming that the feeding factor α is one and $p > 1$, the temperature at which Equation 5 in the main text goes through a minimum simplifies to

$$T_{\min} = \frac{\Delta E/p}{k_{\mathrm{B}} \ln\left[1 + \left(\frac{g_2 k_{\mathrm{nr}}(0)}{k_{\mathrm{r},1}}\right)^{1/(p-1)}\right]} \tag{S2}$$

Solving this for $k_{\mathrm{r},1}$ gives

$$k_{\mathrm{r},1} = k_{\mathrm{nr}}(0) g_2 n^{p-1} \tag{S3}$$

which implies that the intensity ratio reaches a minimum when the absorption rate of the higher-order phonons $p - 1$ is equal to $k_{\mathrm{r},1}$.
